# Evaluating the Clinical Relevance of Codon 594 (G>A) Polymorphism of Estrogen Receptor Alpha in Knee Osteoarthritis

**DOI:** 10.5704/MOJ.1403.016

**Published:** 2014-03

**Authors:** T Tawonsawatruk, P Mulpruek, DF Hamilton, W Wajanavisit, S Tan

**Affiliations:** Department of Orthopaedics, Faculty of Medicine, Ramathibodi Hospital, Mahidol University, Bangkok, Thailand; Department of Orthopaedics, Faculty of Medicine, Ramathibodi Hospital, Mahidol University, Bangkok, Thailand; Department of Orthopaedics, Faculty of Medicine, Ramathibodi Hospital, Mahidol University, Bangkok, Thailand; Department of Orthopaedics, Faculty of Medicine, Ramathibodi Hospital, Mahidol University, Bangkok, Thailand; Department of Radiology, Sarawak General Hospital, Kuching, Malaysia

## Abstract

It has been reported that oestrogen receptor alpha (ER-α) polymorphisms are associated with knee osteoarthritis (OA). In this study, we assessed whether there was any association between the codon 594 (G>A) polymorphism in ER-α and radiographic features of OA or patient function. Radiographs, WOMAC score and patient reported time of symptom onset were assessed in 194 patients presenting for total knee replacement at Ramathibodi hospital over a one year period. ESR-1 genotyping was assessed. There were 107 (55.15%) patients with common homozygote (GG), 78 (40.20%) patients with heterozygote (GA) and nine (4.65%) patients with rare homozygote (AA). There was poor correlation (r = <0.2) between group difference in the radiographic parameters, time of onset of symptom , or in WOMAC scores. This polymorphism is not associated with the clinical features of knee osteoarthritis. The role of this polymorphism is unlikely then to be used as a biological marker predicting the progression of knee OA.

**Key Words:**

Oestrogen receptor alpha polymorphism, Knee osteoarthritis, Radiographic feature, Functional score

## Introduction

Osteoarthritis (OA) is a leading cause of physical disability and is estimated to affect around 40% of people over 70 years of age^1^. Knee OA in particular is a major cause of morbidity and is the primary diagnostic indication for total knee replacement^2^, the volume of which continues to grow unabated globally. Knee OA is characterised by joint space narrowing, osteophyte formation and subchondral sclerosis which manifest primarily as joint pain^3^.

The aetiopathogenesis of knee OA is multifactorial with various independent risk factors having been identified ^3-5^. The role of genetic factors has gained increasing research prominence in recent years, with studies reporting potential factors 6-8. Various single nucleotide polymorphisms (SNPs) such as growth differentiation factor 5 (GDF5), prostaglandin-endoperoxide synthase 2 (PTGS2), and cartilage oligomeric matrix protein (COMP) have been identified in both candidate gene and genome-wide association studies ^9-13^. A particularly interesting association is with the oestrogen endocrine system, where enhanced oestrogen levels have been suggested as being protective in epidemiological studies ^14-16^, and there is optimism that pharmacological interventions such as hormone replacement therapy may moderate the presentation of OA.

The oestrogen receptor alpha (ESR-1) gene encodes the ERalpha receptor found in articular cartilage ^14^. We and others ^13,17-19^ have previously reported polymorphism of this gene to be associated with knee OA in various Asian and European populations. The link to female OA was self-evident and particularly relevant to Asian orthopaedic practice, where there is a distinct gender split in the presentation of knee OA, and typically 75% of knee replacements are carried out in females. A European study of knee OA reported however that ESR-1 polymorphism is associated with radiographic changes and osteophytosis in both males and females ^17^. The mechanism for this increased OA risk with the ESR-1 polymorphism is not yet clearly defined, though Bergink et al. speculate that the polymorphism may be associated with modulation in bone metabolism during adolescence and linked to an increased susceptibility to OA in later life as a result of loading changes to the joint ^17^. Though the link between ESR-1 and osteoarthritis is established, the relationship between this polymorphism and the severity of patient symptoms has not been previously described.

The aim of this study was to examine the association between the codon 594 (G>A) polymorphism in the ESR-1 gene and the radiographic appearance and clinical function scores of patients with clinically defined osteoarthritis.

## Materials and Methods

### 

**Study Population**Data was obtained from patients attending the orthopaedic outpatient assessment clinic of a single university teaching hospital for consideration of total knee replacement. All patients presenting over a single calendar year were invited to participate. Local ethical committee approval was obtained and 194 patients consented to take part in the study. All participants had diagnosis of knee osteoarthritis (based on the American College of Rheumatology criteria ^20^).

**Genotyping**Genotyping was carried out using 5ml of peripheral blood and the polymerase chain reaction restriction fragment length polymorphism (PCR-RFLP) technique; detailed methodology of which has been described previously ^13^. Briefly, the blood was processed for SNP analysis and genomic DNA extracted. PCR primers “GTGGAGGAGACGGACCAAA “(forward) and “TGGCCACTCATCTAGAAAGCC” (reverse) were used to amplify exon 8 of the ER-alpha gene. PCR product was incubated at 37°C with 3 units of BtgI for 4 hours as per the manufacturer’s recommendations (New England Biolabs, Ipswich, MA). The digested product was electrophoresed on 2% agarose gel, stained with ethidium bromide and imaged on an UV transilluminator. The expected fragment length was 101 and 146 bp in GG, 101, 146, and 247 bp in AG, and 247 bp in AA genotypes.

**Assessments of knee osteoarthritis**Patients were assessed in the outpatient clinic prior to any surgical intervention. Demographics (age, gender and BMI), patient reported age of onset of OA symptoms and Western Ontario and McMaster Universities (WOMAC) score ^21^ were assessed at this visit. Radiographs were taken at a separate visit, as per local protocol, within 1 month of the date of patient recruitment.

Anteroposterior and lateral radiographs of the knee were reviewed and graded according to the Kellgren and Lawrence (KL) scores ^22^. This is a 0-4 scale where 0, represents no features of osteoarthritis; 1, is doubtful of osteoarthritis, with minute osteophytes of doubtful importance; 2, minimal osteoarthritis, with definite osteophytes but unimpaired joint space; 3, moderate osteoarthritis, with osteophytes and moderate diminution of joint space; and 4, severe osteoarthritis, with greatly impaired joint space and sclerosis of subchondral bone.

The presence of osteophytes and joint space narrowing was additionally classified (separately to KL score) using a categorical scale (0-2): where 0 represented no features of osteophyte formation or no presence of joint space narrowing; 1, represented marginal osteophyte formation or asymmetrical joint space narrowing, and 2, represented large osteophyte formation or significant joint space narrowing. All images were assessed independently by two consultant orthopaedic surgeons, then consensus of score agreed upon, blinded to patient age, sex and genotype.

**Statistical analysis**Genotype distribution was assessed with the Hardy- Weinberg Equilibrium test, employing an online tool from the Online Encyclopaedia for Genetic Epidemiology studies ^23^. The data were analysed using SPSS 16.0 (Chicago, IL). Correlation of genotype (GG, AG and GG) and radiographic parameters, age of onset and WOMAC score were analysed using the Spearman's rank correlation co-efficient. Between group differences were assessed using analysis of variance (ANOVA). Statistical significance was accepted at p <0.05.

## Results

**Patient characteristics and genotype distribution** whom 24 (12.4%) were male and 170 (87.6%) female. The mean age of patients was 67.41 years (SD= 8.24) and the mean age of onset of symptoms was 59.57 years (SD=9.00). Mean body mass index (BMI) of the individuals participated in the study was 27.10 (SD= 4.19). There were no differences in patient characteristics found between the groups (Table I).

Very few patients carried the AA genotype in ESR-1 polymorphism (Table 1). 55.15% patients carried the common homozygote (GG), 40.20% the heterozygote (GA), and 4.65% the homozygote (AA). The distribution of genotype was considered normal using the Hardy-Weinberg Equilibrium.

**Association between ESR-1 polymorphism and radiographic features**Radiographic scores for the three groups are summarised in Figure 1. KL scores in patients who had genotype AG and AA were slightly higher than in the wild type (GG) group, though this was not statistically significant (ANOVA, p=0.60). The patients with the A allele had more osteophytes; however this was not related to the presentation of the joint space. Radiographic parameters were not significanlty different between genotypes in either osteophyte presence (ANOVA, p = 0.32) or joint space narrowing (ANOVA, p = 0.84).

Further, poor correlation was seen between polymorphism of estrogen alpha receptor with any of the radiographic parameters; KL score, r = 0.12 (p = 0.22), Osteophyteosis r = 0.15 (p = 0.13), or joint space narrowing, r = -0.02 (p = 0.81).

**Association between ESR-1 polymorphism and patient symptoms** The mean age of patient reported symptom onset was broadly the same across the three genotypes (Table I). There were no significant differences between patients who carried the AG, AA and GG genotype (ANOVA, p = 0.99). There was also very poor correlation between genotype of polymorphism and age of symptom onset (r = -0.03, p = 0.71).

WOMAC scores were 57.58 (7.48), 56.20 (3.9) and 60.23 (8.98) for AG, AA and GG groups respectively (Figure 2). There was no significant difference between mean WOMAC score (ANOVA, p = 0.24). Again there was very poor correlation between ERS-1 polymorphism and WOMAC score (r = 0.17, p = 0.08).

## Discussion

The polymorphism of the ESR-1 gene in codon 594 (G>A) is not associated with radiographic changes or with patient symptoms of knee osteoarthritis in patients presenting for total knee replacement. No differences were seen in age of symptom onset, in radiographic severity or reported pain and function (WOMAC score) in patients with AA, AG or GG genotypes.

Previous reports have highlighted ESR-1 polymorphism as a risk factor of knee osteoarthritis. In a European study of repeat polymorphisms, ESR-1 was found to be associated with idiopathic knee OA in a case-control cohort design of 158 patients ^24^. Similarly Asian studies have reported an association between PvuII and XbaI SNPs in intron 1 of the ER-alpha gene and an increase in the prevalence of generalized OA in 383 Japanese women ^18^ and ER-alpha codon 594 G/A has been highlighted as a risk factor for knee OA in the Korean and Thai population ^13, 19^.

The Rotterdam Study (of 1483 individuals), using direct molecular haplotyping to determine the relationship between 2 polymorphisms in the ERα gene demonstrated a significant association with the presence of osteophytes in Knee OA^17^. Our data suggests that patients with the A allele presented with slightly worse radiographic scores, however no statistically significant differences were observed between groups. Similarly a case control study from Spain suggested no correlation between six separate polymorphisms in GDF5, PTGS2, 7q22 locus, DVWA, DIO3, and ASPN and the onset of knee OA. They suggested simply that old age and genetic factors are independent risk factors of knee OA^25^.

The WOMAC index is self-administered questionnaire that is widely used to quantify the functional ability of patients with OA and following knee replacement. It can be used to monitor disease progression and compare treatments. Our study found no differences in the patients WOMAC score between A and G alleles. We believe this is the first study to assess the association between ERS-1 polymorphism and patient function using the WOMAC index. The only previous genetic study assessed the association between pain perception and nucleotide polymorphisms in the SCN9A gene. This study suggested that patients who carried the minor allele had higher WOMAC pain scores ^26^.

**Strengths and limitations**Despite assessing almost 200 individuals, it is possible that our study was underpowered to detect a difference. However that less than 5% of patients presenting for consideration of OA carried the polymorphism in question suggests a very poor clinical relevance of this particular factor.While we assume that the results are generalizable to the wider population, the primary limitation of this study is the potential selection bias as sample data was obtained from patients presenting to a single university hospital. This limitation is mitigated by the additional radiographic and functional evaluation that we were able to perform with this study design. The distribution of patient genotype was considered normal using the Hardy-Weinberg Equilibrium, thus it is unlikely that selection, mutation, migration, or genetic drift have effect allele frequencies in our study group27 lending to the validity of our findings.

**Table I: Demographic data by ESR-1 genotype:**
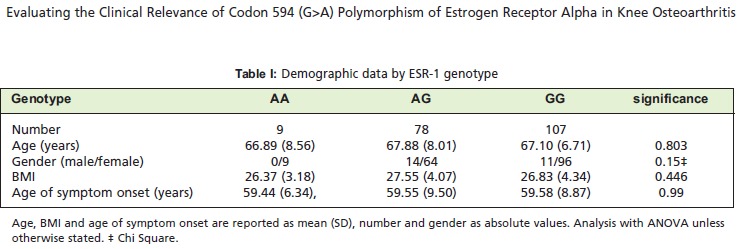


**Figure I: Radiographic parameters: The graphs show the mean scores on (a) KL score, (b) osteophyte formation (c) joint space narrowing (Error bar, +/-2SD) from the OA patient with different genotypes (GG, AG and AA).**
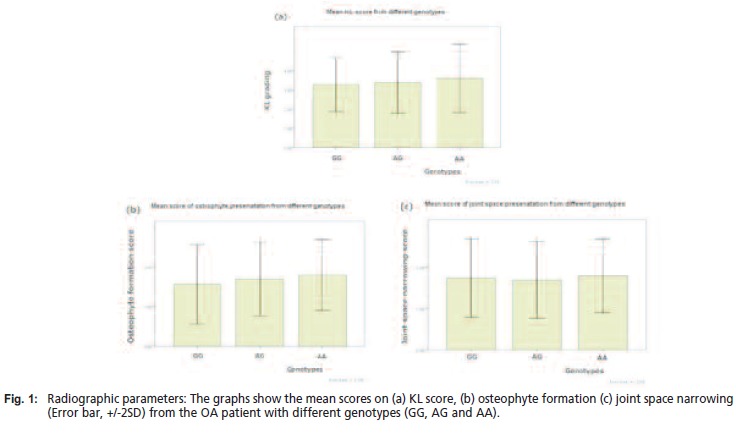


**Figure II: WOMAC score: The graph show the mean WOMAC score from the OA patient with different genotypes (GG, AG and AA). (Error bar, +/-2SD).**
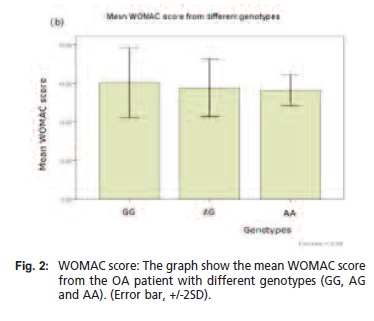


## Conclusion

Though ESR-1 polymorphism has been previously linked with the development of knee osteoarthritis, we found no correlation with disease severity, clinical features or radiographic appearance in patients presenting for total knee replacement by allele differences. In addition, the very small numbers of individuals in our study that carried this polymorphism suggests that this individual genetic factor has minimal clinical relevance to the treatment of end-stage knee OA and it is unlikely to influence the progression of patients presenting with knee OA. Our results suggest it is unlikely that single polymorphisms will account for the clinical presentation of knee osteoarthritis. Further studies on genetic risk factors or polymorphisms related to knee OA should then perhaps focus on assessing multiple target loci. Multicentre (and preferably multinational) studies would also be useful to help explain the genetic risk factors relating to knee OA.
